# Supplementary material for: Chromothripsis during telomere crisis is independent of NHEJ, and consistent with a replicative origin
Source: Genome Res. 2019 May;29(5):737–49. doi: 10.1101/gr.240705.118 (PMC6499312; doi:10.1101/gr.240705.118)
Supplement: Supplemental Material [file supp_gr.240705.118_Supplemental_file_1.zip › contigs/annotated_contigs/DB51/contig.2.DB51_length_361_mean_cov_2.90858725762.docx]

**DB51_length_361_mean_cov_2.90858725762**

```
TTTGCACTCCCAAATGGGGGACTTGGTGTGCTTGTTTCAGAGAGAGTGTAGGAGGAGGAAAAGAAGGGAAATGACTCCACATCTCAGCG
 >chr10:30383529-30383861 - E=4e-189
AGATCAGGGAGACAGATATTCGGGAGGAGGTGGGAGAATGAAAATGACTGGGAACAGGTGGGCTAAATCGGTGAGGTGTGTGGGGAAGA

AGCTAAGTTAGGAGTGGGCGGCTGGTTTTTCCAAAGGCAAAGATGGTGTCTTAGTTGGGAGCAGGAAGAGAACTGATTTCTTTCCATAA

GGCTGCCATGCAGTCTCCTCCAGACTGCATAAGGGAAAATGCAGGTAGAAGTAGCACTCGTCTGC|A|TGGCAAACTCATTGGTATGGT
                                                                   >chr18:67127879-6712
AAATTTA7907 +
```
